# Cancer and Involuntary Weight Loss: Failure to Validate a Prediction Score

**DOI:** 10.1371/journal.pone.0095286

**Published:** 2014-04-24

**Authors:** Cristian Baicus, Mihai Rimbas, Anda Baicus, Simona Caraiola, Grupul de Studiu al Scaderii Ponderale Involuntare

**Affiliations:** 1 Colentina University Hospital, Departments of Internal Medicine and Gastroenterology, Bucharest, Romania; 2 Clinical Research Unit, Réseau d' Epidémiologie Clinique International Francophone, Bucharest, Romania; 3 Carol Davila University of Medicine and Pharmacy, Bucharest, Romania; 4 I. Cantacuzino National Institute of Research and Development in Microbiology-Immunology, Bucharest, Romania; University Hospital Lausanne, Switzerland

## Abstract

**Background:**

Many patients who have involuntary weight loss have cancer. The Hernandez prediction rule includes 5 variables (elevated levels of alkaline phosphatase and lactate dehydrogenase, low albumin, high white blood cell count, and age >80 years). The purpose of this study was to evaluate the validity of the prediction rule.

**Methods:**

We prospectively evaluated 290 consecutive inpatients and outpatients who had involuntary weight loss. Clinical, hematologic, and biochemical parameters were determined. There were 259 patients who had follow-up at 6 months to determine the cause of involuntary weight loss, and 31 other patients were lost to follow-up. The 5 variables were introduced into a regression logistic model with cancer as a dependent variable.

**Results:**

Cancer was diagnosed in 72 of the 290 patients (25%) who had involuntary weight loss. Bivariate analysis showed that serum albumin, C-reactive protein, erythrocyte sedimentation rate, alkaline phosphatase, iron, lactate dehydrogenase, white blood cell count, hemoglobin, and ferritin levels were associated with cancer (range of area under the receiver operating characteristic curve, 0.589 to 0.688). Multivariate analysis showed that albumin, erythrocyte sedimentation rate, iron, white blood cell count, and lactate dehydrogenase levels were associated with cancer. When dichotomized, only low albumin (odds ratio, 2.6, CI [1.3–5.2]) and high alkaline phosphatase (odds ratio, 2.3, CI [1.7–4.7]) were associated with cancer. The area under the receiver operating characteristic curve of the 5-variable prediction rule was only 0.70 (95% confidence interval, 0.61–0.78). The negative predictive value of this model with 3 variables (age >60 y, alkaline phosphatase, and albumin level) increased from 85% to 95% when all tests were negative.

**Conclusions:**

In patients who had involuntary weight loss, those who have cancer are likely to have ≥1 abnormal laboratory test. The 5-variable prediction rule had a significantly lower accuracy than originally reported. Further evaluation of the 3-variable modification of the prediction rule may be useful.

## Introduction

The frequency of involuntary weight loss (IWL) is 5% in patients who are admitted to internal medicine departments [1], [2]. Cancer is diagnosed in 25% patients who have IWL [1]. The history and physical examination may provide important information to help determine the cause of IWL, but in some patients the cause of IWL of unknown origin may be difficult to determine. Most patients who have IWL do not have cancer, and 30% patients who have IWL have psychiatric disorders or IWL of unidentified cause [3]. However, it is important to determine whether or not the patient has a life threatening disease such as cancer.

A study of 101 patients who had IWL showed that the probability of having cancer was unlikely when the evaluation had normal testing including physical examination, complete blood count, C-reactive protein (CRP), aspartate aminotransferase (AST), alanine aminotransferase (ALT), alkaline phosphatase (ALP), lactate dehydrogenase (LDH), albumin, ferritin, chest radiography, and abdominal ultrasound; all 22 patients who had cancer had at least one abnormal laboratory test [4]. However, 50% patients who did not have an organic diagnosis had ≥1 laboratory abnormality [4]. Age, anemia, and erythrocyte sedimentation rate (ESR) may help predict whether a patient has IWL associated with cancer; in patients who have IWL, the probability of having cancer is 64% in patients aged >62 years who have anemia and high erythrocyte sedimentation rate (ESR) but only 9% in patients younger than 63 years who have normal hemoglobin and ESR [3].

The Hernandez score is a prediction score to identify cancer. The score was retrospectively developed in 256 patients who had unexplained IWL and was validated prospectively in 52 patients, at the same site [5]. The independent predictors of cancer included elevated alkaline phosphatase level, elevated LDH level, high white blood cell count, low albumin level, and age >80 years [5]. This prediction score is cited in several point-of-care clinical sources, UpToDate [6], Essential Evidence Plus [7] and First Consult. A clinical calculator of cancer risk is available from Essential Evidence Plus [7].

The purpose of the present study was to validate externally the Hernandez score.

## Materials and Methods

### Setting and patients

We prospectively studied adult patients referred for IWL, with unknown origin despite clinical assessment, who were admitted as inpatients or referred to the day hospital in the Department of Internal Medicine of Colentina University Hospital, Bucharest, Romania. All consecutive patients (age, ≥18 y) were included when they had either (1) documented IWL ≥5% body weight within the previous 6 months or (2) declared “very much” or “much” concern about the amount of weight loss (defined by a Likert scale with 5 levels: 5, very much; 4, much; 3, average; 2, little; or 1, not at all). The latter inclusion criterion was applied only to select patients who had no baseline weight documentation and for whom the amount of weight loss could not be computed to fulfill the first inclusion criterion; for these patients, it was required that the presence of weight loss was confirmed either by a relative or changes in the size of clothing or belts. Patients were excluded for voluntary weight loss, known malignancy, or availability of clinical diagnostic information that enabled the diagnosis of the cause of weight loss. The study was conducted according to the guidelines of the Declaration of Helsinki. The study protocol was approved by the Colentina Hospital ethics committee, and all patients agreed to participate and gave informed consent before enrolling.

### Study design

The diagnostic evaluation was not standardized, but was determined by each participating physician and varied with the clinical and laboratory information available for each patient. Consistent with the objectives of the study, all patients on admission had testing that included a complete blood cell count and determination of blood ESR, serum CRP, iron, albumin, ALP, ALT, LDH, and ferritin levels. Tumor necrosis factor α, interleukin 1β, and interleukin 6 levels, and red cell distribution width, were determined for other objectives of the research project reported elsewhere [8]–[10]. All variables were recorded by every physician in a preformed questionnaire and entered by 1 author into the statistical software (SPSS) database. Patients were followed for 6 months (173 by follow-up visit, 86 by telephone calls with patient), and the final diagnosis was recorded to avoid misclassification about presence or absence of cancer.

### Laboratory procedures

The complete blood cell count was determined with an analyzer (Sysmex XT 1800i counter, Sysmex Corporation, Kobe, Japan). Serum albumin, iron, ALP, ALT, LDH, and CRP levels were measured (Cobas 6000 Modular P 800 analyzer, Roche Diagnostics, Rotkreuz, Switzerland). The ESR was determined with the Westergren method [11]. All tests were performed at the central laboratory of the hospital except ferritin, which was measured (Chemwell 2910 analyzer, Awareness Technology, Palm City, FL, USA) at the Cantacuzino Institute of Research in Microbiology and Immunology, Bucharest. The quality of results was validated throughout the study by regular internal quality control procedures and participation in an external quality assessment program.

### Sample size

It was estimated that ≥50 patients who had cancer were needed for multiple logistic regression because the model of Hernandez had 5 variables and ≥10 outcome events (patients who had cancer) were necessary for every independent variable in the model [12]. The prevalence of cancer in recent IWL studies was 22% to 38% [3]–[5], [13]. Therefore, we calculated that ≥250 patients who had IWL should be included for a worst case prevalence of 20%.

### Statistical analysis

Data analysis was performed with statistical software (Stata 11, StataCorp, College Station, TX, USA; and SPSS 16.0, SPSS, Inc., Chicago, IL, USA). An Internet-based calculator (EBM calculator 1.0, www.cebm.utoronto.ca) was used for calculation of sensitivity, specificity, predictive values, and likelihood ratios. The outcome was the diagnosis of cancer as the cause of IWL, and the predictor variables included the clinical variables (age, sex, amount of weight loss, and smoking) and laboratory variables recorded. Categorical variables were reported as frequency and analyzed by Fisher exact test. Continuous variables that were not normally distributed were reported as median (minimum to maximum) and analyzed with Mann-Whitney test, Kruskal-Wallis test, or Kendall τ (tau) rank correlation. Receiver operating characteristic (ROC) curves were generated; areas under the curve (AUC) and 95% confidence intervals (CI) were determined.

The variables associated with cancer in bivariate analysis were evaluated with a logistic regression model. For validation of the model of Hernandez [5], we dichotomized the variables using the same criteria for cutoff values, the normal limits of our laboratory for white blood cell count, serum ALP, LDH, and albumin levels (white blood cell count >12×10^9^/L (12 000/µL), albumin <3.5 g/dL, ALP >104 U/L, and LDH >220 U/L), and we used the cutoff age 80 years. The variables were introduced into the logistic regression model, and AUC values were calculated. A sensitivity analysis was performed by fitting the prediction model only in the subsample of patients who had known amount of weight loss. Age >80 years was not statistically associated with cancer in bivariate or multivariable analysis; therefore, the age cutoff was changed to age >60 years and a new logistic regression model was evaluated with 3 variables (age >60 y, low serum albumin level, and high ALP level), the AUC was calculated, and the positive and negative predictive values with 95% CI were calculated [14]. The variables were selected for logistic regression with the enter method (all studied variables were included, without any sequential selection) [12]. Hypothesis testing was 2-tailed. Statistical significance was defined by *P*<.05.

## Results

### Patients

**Figure 1:**
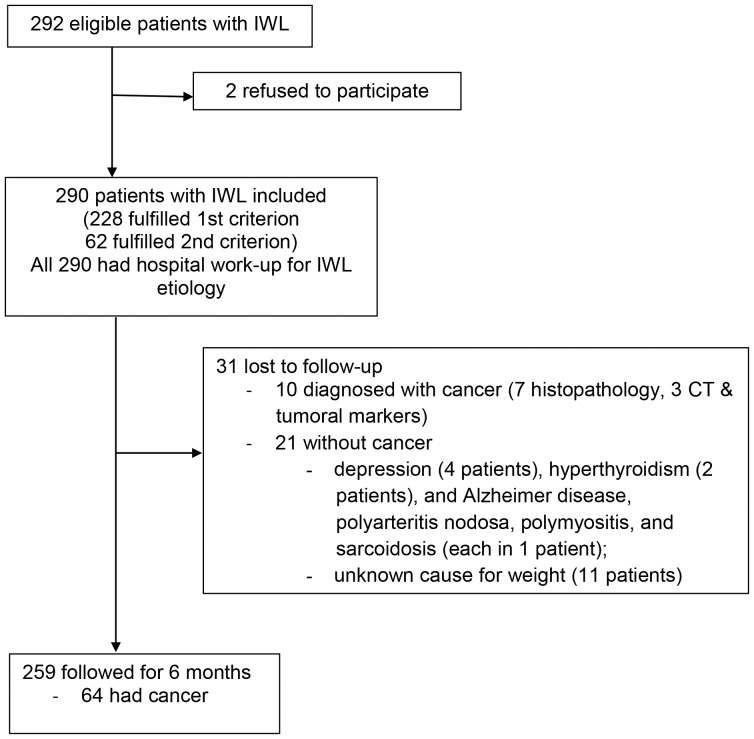
Flow Diagram For the Study of Patients Who Had Involuntary Weight Loss (IWL). The first inclusion criterion was documented IWL ≥5% body weight within the previous 6 months. The second inclusion criterion was a declared “very much” or “much” concern about the amount of weight loss (defined by a Likert scale with 5 levels: 5, very much; 4, much; 3, average; 2, little; or 1, not at all). ***Abbreviation:*** CT, computed tomography.

From January 2009 to March 2010, there were 290 consecutive patients who had IWL of unknown cause and were included in the study (Figure 1). In the 228 patients who had known amount of weight loss (first inclusion criterion), there was a modest correlation between the measured weight loss and the patient's estimate of weight loss on the Likert scale (Kendall τ: r, 0.509; *P*≤.001) (Figure S1). We assumed that the same correlation was present for the 62 patients who had unknown baseline weight. The patients with IWL had median age 67 years (range, 22 to 94 y), and 146 patients (50%) were men. The patients who had cancer had median age 69 years (range, 44 to 93 y), and 42 (58%) were men.

259 patients (89%) had follow-up for 6 months to diagnose or exclude the diagnosis of cancer that was not identified during hospitalization. There were 31 patients (11%) who were considered lost to follow-up because they did not complete evaluation at 6 months (cancer, 10 patients; no known cancer, 21 patients [other conditions, 10 patients; unknown cause of weight loss, 11 patients]) (Figure 1).

### Outcome

Cancer was diagnosed in 72 of the 290 patients who had IWL (25%). Other diagnosed causes of IWL included infectious or inflammatory diseases in 51 patients (18%), psychiatric diseases in 49 patients (17%), and other chronic organic diseases in 96 patients (33%). No cause of IWL was identified in 22 (8%) patients. In 66 patients who had gastrointestinal diseases, 35 patients (12%) had digestive nonneoplastic diseases and 31 patients (11%) had cancer (gastrointestinal cancer, 22 patients [8%]; pancreas, liver, or biliary cancer, 9 patients [3%]). Most patients who had cancer had ≥1 abnormal hematologic or biochemical parameter; only 1 patient who had urinary bladder cancer had all blood tests normal but had microscopic hematuria.

Bivariate analysis showed that serum albumin, CRP, ESR, ALP, iron, LDH, white blood cell count, hemoglobin, and ferritin levels were associated with cancer (Table 1). Age, sex, amount of weight loss (absolute, percent, or Likert scale), smoking, and ALT level were not associated with cancer (data not shown). There was no relation between cancer type and amount of weight loss (absolute or percent weight loss) (Figure S2). In multivariable analysis (quantitative variables), only albumin, ESR, iron, white blood cell count, and LDH levels were associated with cancer.

**Table 1:** Bivariate Analysis For Variables That Were Significantly Associated with Cancer in Patients Who Had Involuntary Weight Loss*.

Variable	Patients Without Cancer (n = 218)	Patients With Cancer (n = 72)	*P*≤	Area Under Curve (95% Confidence Interval)
Albumin (g/dL)	3.98 (2–5.2)	3.6 (2.1–6.1)	.001	0.691 (0.618–0.765)
C-reactive protein (mg/L)	7 (51–328)	43.6 (1.1–550)	.001	0.688 (0.613–0.763)
Erythrocyte sedimentation rate (mm/h)	38.5 (2–140)	56 (3–140)	.001	0.666 (0.597–0.735)
Alkaline phosphatase (U/L)	85 (10–1224)	100 (41–919)	.001	0,666 (0.591–0.741)
Iron (µg/dL)	53.5 (13–162)	37 (3.9–162)	.002	0.632 (0.550–0.713)
Lactate dehydrogenase (U/L)	194 (91–927)	220 (26–2100)	.002	0.625 (0.541–0.709)
White blood cell count (/µL)	7380 (1580–21 800)	9370 (3592–102 000)	.007	0.606 (0.526–0.687)
Hemoglobin (g/dL)	11.76 (6.1–16.4)	11.6 (7.2–14.7)	.02	0.593 (0.518–0.669)
Ferritin (ng/mL)	102.5 (3–2000)	191 (12–1000)	.03	0.589 (0.513–0.666)

*Data reported as median (minimum to maximum).

### Evaluation of the prediction rule

All variables from the model of Hernandez that were associated with cancer were quantitative variables; age was the only quantitative variable that was not associated with cancer (Table 1). This was confirmed with analysis of ROC curves for the variables (Figure S3).

When dichotomized, in bivariate analysis, age dichotomized at 80 years was not significantly associated with cancer, but tests that were significantly associated with cancer were high white blood cell count(*P*≤.001), low albumin (*P*≤.001), and high ALP (*P*≤.001) and LDH (*P*≤.04). These parameters performed only modestly as diagnostic tests for cancer (Table 2).

**Table 2:** Components of the Hernandez Model as Diagnostic Tests for Cancer in Patients Who Had Involuntary Weight Loss*.

Variable	Sensitivity	Specificity	Positive Likelihood Ratio	Negative Likelihood Ratio	Positive Predictive Value	Negative Predictive Value
White blood cells >12 000/µL	0.25	0.90	2.49	0.83	0.45	0.78
Serum alkaline phosphatase >220 U/L	0.50	0.78	2.28	0.64	0.43	0.83
Serum albumin <3.5 g/dL	0.41	0.80	2.08	0.73	0.39	0.81
Serum lactate dehydrogenase >104 U/L	0.44	0.70	1.47	0.8	0.32	0.79

*Age >80 years was not reported because it was not associated with cancer in bivariate analysis.

**Figure 2:**
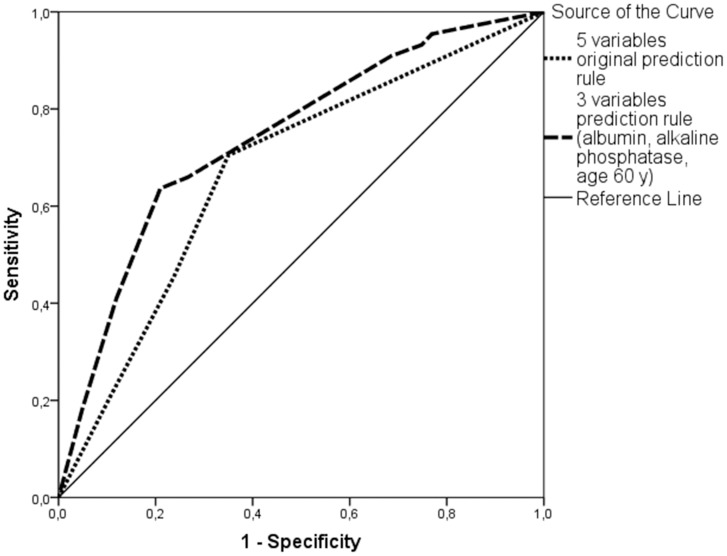
Receiver Operating Characteristic Curves of the Hernandez and Present Models in Patients Who Had Involuntary Weight Loss. The model of Hernandez had 5 variables, and the present modified model had 3 variables.

In multivariable analysis with these variables dichotomized with all patients, only low albumin (OR = 2.6, CI [1.3–5.2]), and high ALP (OR = 2.3, CI [1.7–4.7]) were associated with cancer (Table 3). The AUC of the prediction rule was 0.70 (95% CI, 0.61–0.78) in the analysis with all patients (Table 3 and Figure 2). A repeat analysis with the 228 patients who had known weight loss gave similar results (Table 3). Comparison of the odds ratios of the variables in the previous [5] and present study showed that fewer variables had significance in the present than previous study (Table 3). The negative predictive value of the model (when all tests were negative) was 85%.

**Table 3:** Multivariable Analysis in Patients Who Had Involuntary Weight Loss and Comparison with Hernandez Study*.

Variable	All patients (290 patients)			Patients included by the first criterion (known amount of weight loss) (228 patients)			Hernandez study [5]
	Odds Ratio	(95% Confidence Interval)	*P*	Odds Ratio	(95% Confidence Interval)	*P*	Odds Ratio
Age >80 y	1.2	(0.4–3.7)	.82	0.8	(0.2–3.0)	.69	3.4
High white blood cell count	2.2	(0.9–5.1)	.07	1.8	(0.7–4.7)	.21	3.6
Low serum albumin	2.6	(1.3–5.2)	.02	2.5	(1.1–5.6)	.02	6.7
High serum alkaline phosphatase	2.3	(1.2–4.7)	.01	2.1	(1.0–4.2)	.04	12
High serum lactate dehydrogenase	1.3	(0.6–2.5)	.53	1.3	(0.6–2.8)	.48	12.5
AUC	0.70 (0.61–0.78)			0.70 (0.61–0.80)			0.89 (0.87–0.91)

*Logistic regression. ***Abbreviations:*** AUC, area under the curve.

When the age cutoff was changed from 80 to 60 years, age was associated with cancer in bivariate (*P*≤.002) and multivariable analysis (*P*≤.002, Table 4), and the negative predictive value of this model with 3 variables (age >60 y, ALP, and albumin level) increased to 95% when all tests were negative (Table 5).

**Table 4:** Multivariable Analysis in Patients Who Had Involuntary Weight Loss*.

Variable	Odds Ratio	(95% Confidence Interval)	*P*
Age (>60 y)	5.31	(1.88–15.02)	.002
Serum albumin (<3.5 g/dL)	2.44	(1.14–5.23)	.03
Serum alkaline phosphatase (>104 U/L)	2.67	(1.23–5.80)	.02

*N = 290 patients. Age cutoff, 60 years. Area under the receiver operating characteristic curve for the logistic regression model in the entire patient group: 0.74; 95% confidence interval, 0.66–0.81.

**Table 5:** Modified Regression Model For the Relation Between Clinical Variables and Probability of Having Cancer in Patients Who Had Involuntary Weight Loss*.

-----	----Variable---	-----	Probability	of
Age >60 y	Alkaline Phosphatase >104 U/L	Albumin <3.5 g/dL	Having Cancer (%) (95% Confidence Interval)	Not Having Cancer (%) (95% Confidence Interval)
No	No	No	5 (3–8)	95 (92–97)
No	No	Yes	11 (8–14)	89 (86–92)
No	Yes	No	13 (10–17)	87 (83–90)
Yes	No	No	20 (16–24)	80 (76–84)
No	Yes	Yes	25 (21–30)	75 (70–79)
Yes	No	Yes	34 (30–39)	66 (61–70)
Yes	Yes	No	40 (35–45)	60 (56–65)
Yes	Yes	Yes	59 (54–64)	41 (36–46)

*N = 290 patients.

## Discussion

The present study showed a lower accuracy of the Hernandez cancer prediction model in patients who had IWL (AUC, 0.69) than reported previously (AUC, 0.89) [5], suggesting that the model may not be accurate. The prediction power was increased by excluding white blood cell count and elevated LDH level and changing the age cutoff to 60 years.

Another previous study in 50 patients aged ≥65 years who were admitted for IWL showed that the Hernandez score failed to predict cancer [15]. In that study, all elderly patients who lost weight were included. The present inclusion criteria were similar to previous criteria [5], and we included adult patients who had no known cause of weight loss after the history and physical examination.

The Hernandez score previously was validated with 5 variables on only 52 patients; the present study was performed with a larger group of patients (290 patients). The Hernandez score was considered important, cited in several point-of-care texts, and used by clinicians during the evaluation of patients who had IWL. However, the predictive values were weak in the original study, and they did not clearly exclude or rule-in the diagnosis of cancer. The present study showed that the AUC of the Hernandez score was not adequate to enable selection of a cutoff level for acceptable sensitivity, specificity, or likelihood ratios. The cutoff age of 80 years was too high for our patients and probably was not associated significantly with cancer because we had only 19 patients (7% of whole cohort) aged >80 years. A cutoff age of 60 years was more suitable, was retained in the model, and was a stronger predictor (having the highest odds ratio) for cancer than the other 2 variables (albumin and ALP). This modified model, with only 3 variables (age >60 years, low serum albumin, and high ALP level) had a better negative predictive value when all tests were negative and age <60 y. However, additional evaluation is necessary to validate this modified model.

Limitations of the present study included that we required that patients met 1 of 2 different criteria for inclusion, which could have driven to different populations. However, additional analysis showed that similar proportions of patients were diagnosed with cancer in the group that satisfied the first inclusion criterion (cancer in 57 of 228 patients [25%]) and the second criterion (cancer in 15 of 62 patients [24%]). In addition, the prediction model gave similar results when it was applied to all patients or the subset of patients who had a known amount of weight loss.

We did not identify significant differences in the amount of IWL between different types of cancer, possibly because the sample size precluded analysis of several different types of cancer. Previous studies have not evaluated the association between the amount of IWL and type of cancer [4], [5], [13], [15]–[22].

In summary, in patients who have IWL, the patients who have cancer are likely to have ≥1 abnormal laboratory test [4]. In contrast, a patient aged <60 years who has normal serum albumin and ALP levels may have a low probability that the IWL is associated with cancer. Further study is justified to validate these findings with a larger group of patients.

## Supporting Information

Figure S1 **Relation Between Self-Estimation of Weight Loss and Measured Weight Loss in Patients Who Had Involuntary Weight Loss.**
(DOCX)

Figure S2 **Amount of Weight Loss With Different Types of Cancer in Patients Who Had Involuntary Weight Loss.** (A) Absolute weight loss. (B) Percent weight loss.
(DOCX)

Figure S3 **ROC curves of the separate variables included in the original prediction rule (Hernandez).**
(DOCX)
