# Supplementary material for: Cancer and Involuntary Weight Loss: Failure to Validate a Prediction Score
Source: PLoS One. 2014 Apr 24;9(4):e95286. doi: 10.1371/journal.pone.0095286 (PMC3999093; doi:10.1371/journal.pone.0095286)

Figure S1. Relation Between Self-Estimation of Weight Loss and Measured Weight Loss in Patients Who Had Involuntary Weight Loss.


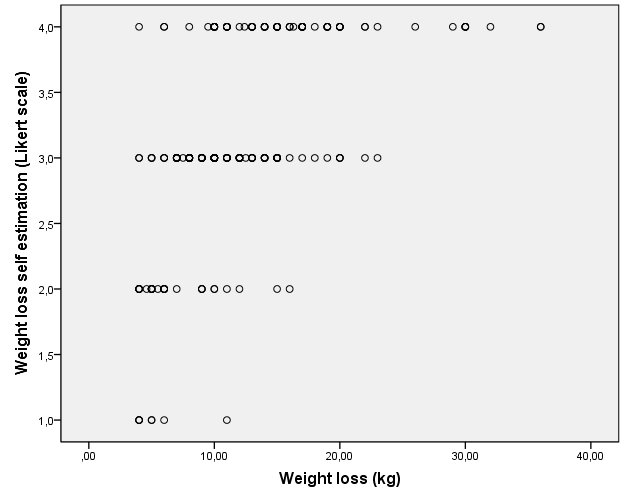

Supplement: Figure S1 — Relation Between Self-Estimation of Weight Loss and Measured Weight Loss in Patients Who Had Involuntary Weight Loss. (DOCX) [file pone.0095286.s001.docx]
