# Supplementary material for: Cancer and Involuntary Weight Loss: Failure to Validate a Prediction Score
Source: PLoS One. 2014 Apr 24;9(4):e95286. doi: 10.1371/journal.pone.0095286 (PMC3999093; doi:10.1371/journal.pone.0095286)
Supplement: Figure S2 — Amount of Weight Loss With Different Types of Cancer in Patients Who Had Involuntary Weight Loss. (A) Absolute weight loss. (B) Percent weight loss. (DOCX) [file pone.0095286.s002.docx]

Figure S2. Amount of Weight Loss With Different Types of Cancer in Patients Who Had Involuntary Weight Loss.


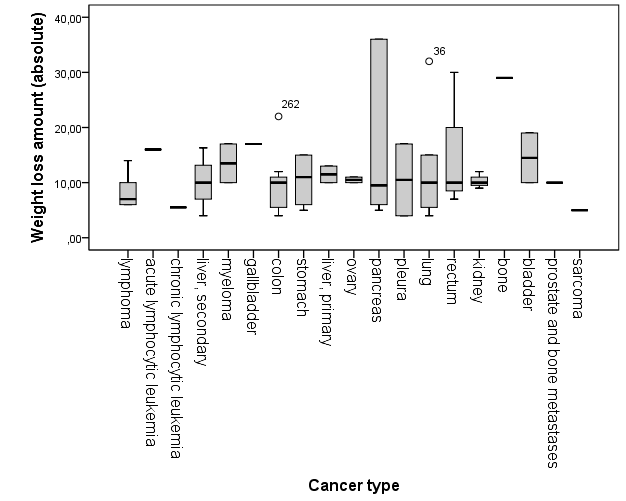


A. Absolute weight loss.


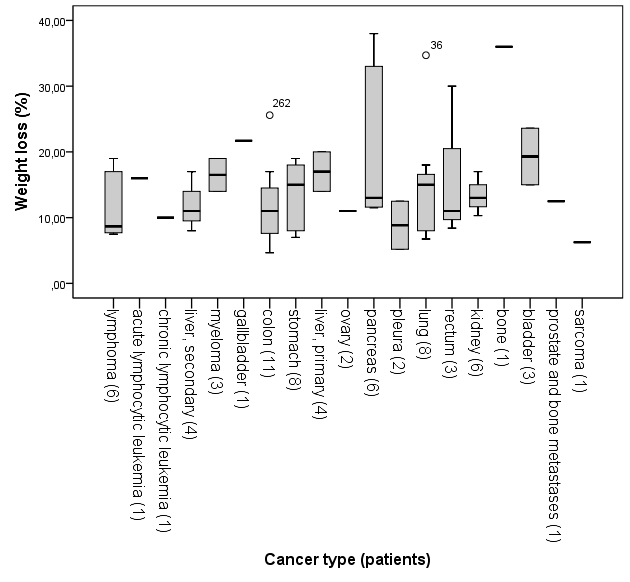


B. Percent weight loss.
