# Supplementary material for: Cancer and Involuntary Weight Loss: Failure to Validate a Prediction Score
Source: PLoS One. 2014 Apr 24;9(4):e95286. doi: 10.1371/journal.pone.0095286 (PMC3999093; doi:10.1371/journal.pone.0095286)

Figure S3. ROC curves of the separate variables included in the original prediction rule (Hernandez)


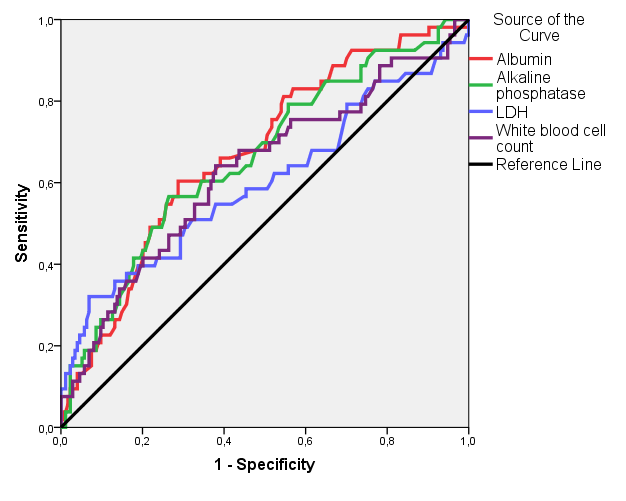

Supplement: Figure S3 — ROC curves of the separate variables included in the original prediction rule (Hernandez). (DOCX) [file pone.0095286.s003.docx]
